# Sensory-Based Identification of Aroma-Active Compounds in Hotpot Seasoning before and after Boiling

**DOI:** 10.3390/molecules26195727

**Published:** 2021-09-22

**Authors:** Mingguang Yu, Suyan Wan, Huanlu Song, Yu Zhang, Chuanming Wang, Hongqiang Wang, Haowen Wang

**Affiliations:** 1 Laboratory of Molecular Sensory Science, College of Food and Health, Beijing Technology and Business University (BTBU), Beijing 100048, China; a1402010411@163.com (M.Y.); ab1102576462@163.com (S.W.); zhang_yu@btbu.edu.cn (Y.Z.); 2 Sichuan Teway Food Group Co., Ltd., Chengdu 610021, China; yfzc_wangchuanming@teway.cn (C.W.); wanghaowen@teway.cn (H.W.)

**Keywords:** hotpot seasoning, SGC/GC×GC-O-MS, boiling, key aroma-active compounds, multivariate statistical analysis

## Abstract

Boiling, the most frequent edible way to hotpot seasoning (HS), exerts a significant impact on the aroma of HS. The present study employed, for the first time, a novel switchable system between GC-O-MS and GC×GC-O-MS (SGC/GC×GC-O-MS) to study the aroma compounds of HS and hotpot seasoning boiling liquid (HSBL). A total of 79 aroma compounds and 56 aroma-active compounds were identified. The aroma extract dilution analysis (AEDA) was used to analyze the differences between the key aroma-active components in the HS and HSBL. The results showed that 13 aroma-active components were significantly affected by boiling, such as D-limonene, methional, and linalool. Moreover, a total of 22 key aroma-active components were identified through the odor activity values (OAVs) calculation. Of them, (*E*)-2-octenal (fatty) and linalool showed a significant difference, suggesting them to be the most critical aroma-active compounds in the HSBL, and HS, respectively. Finally, the correlation between key aroma-active compounds and the sensory properties of HS and HSBL was studied. These results demonstrated that the OAVs of key aroma-active compounds could characterize the real information of samples through bidirectional orthogonal partial least squares (O2PLS). The analysis results were consistent with the sensory evaluation results.

## 1. Introduction

Hotpot has emerged as the popular condiment among consumers worldwide due to its rich taste. The characteristic flavor differs in different hotpot types, which could be attributed to hotpot seasoning (HS). HS, comprising primary and auxiliary materials, is developed through raw materials pretreatment, configuration, and frying. Of them, the primary materials include animal or vegetable oil, salt, chili, bean paste, etc., while the auxiliary ingredients include spices. Therefore, HS is regarded as the core of hotpot, contributing to the quality and flavor of hotpot [1,2]. Moreover, the different constitutes of raw materials in the HS affects the quality and flavor of HS. For instance, butter is a commonly used animal fat in HS, which is rich in minerals, such as fatty acids, vitamins, phospholipids, and other nutrients. Furthermore, butter can contribute to the unique aroma and taste of HS [3], which is inconsistent with the HS prepared from vegetable oil.

Aroma is a main major index to evaluate the quality of food [4]. Therefore, investigating the aroma composition of food at the molecular level is highly necessitated to develop high-quality products. In recent years, flavor analysis has been widely adopted for analyzing HS. Hu et al. [5] analyzed 16 types of HS by solid-phase microextraction (SPME) combined with gas chromatography-olfactory-mass spectrometry (GC-O-MS). A total of 141 volatile compounds were detected, and 52 aroma-active compounds were smelled. Of them, nine aroma-active compounds were common in all samples, including β-myrcene, α-terpene, linalool, linalyl acetate, etc. Sun et al. [1] detected four types of commercially available HS by GC-O-MS using SPME and solvent-assisted flavor evaporation (SAFE) to aroma compounds. The key aroma-active compounds in the butter of HS were determined to be 2-furfuryl alcohol, 2-acetylthiazole, (*E*)-2-decenal, (*E*)-2-nonenal, linalool, D-limonene, anisole, etc. Similarly, Zeng et al. [6] extracted and analyzed the flavor compounds of HS (10 kinds) in the Sichuan and Chongqing regions of China through principal component analysis (PCA) and found that aldehydes, hydrocarbons, ketones, esters, and alcohol compounds constituted the characteristic flavor of HS.

Boiling is the most frequent edible way to use HS. Accumulating studies have reported that the volatile compounds of food ingredients significantly change during the food boiling process, including aroma compounds [7,8]. However, very few have reported changes to the aroma compounds, especially key aroma-active compounds during boiling through GC-MS/GC-O technology. Moreover, the phenomenon of the co-elution compound is often accompanied [9,10] and the matrix of HS is more complex due to the limited capacity of one-dimensional GC. Therefore, this method could not comprehensively analyze the aroma compounds in the HS. Herein, a new switchable system between GC-O-MS and GC×GC-O-MS (SGC/GC×GC-O-MS) [11,12] was used to identify the differences of the volatile compounds in the HS and HSBL. Additionally, the aroma extract dilution analysis (AEDA) and odor activity values (OAVs) were employed to explore the key aroma-active compounds in the samples and their differences among the samples. Finally, the bidirectional orthogonal partial least squares (O2PLS) model was employed to study the relationship between the key aroma-active compounds and sensory properties.

## 2. Results and Discussion

### 2.1. The Effect of Boiling on Aroma Profile Evaluation of Hotpot Seasoning

**Figure 1:**
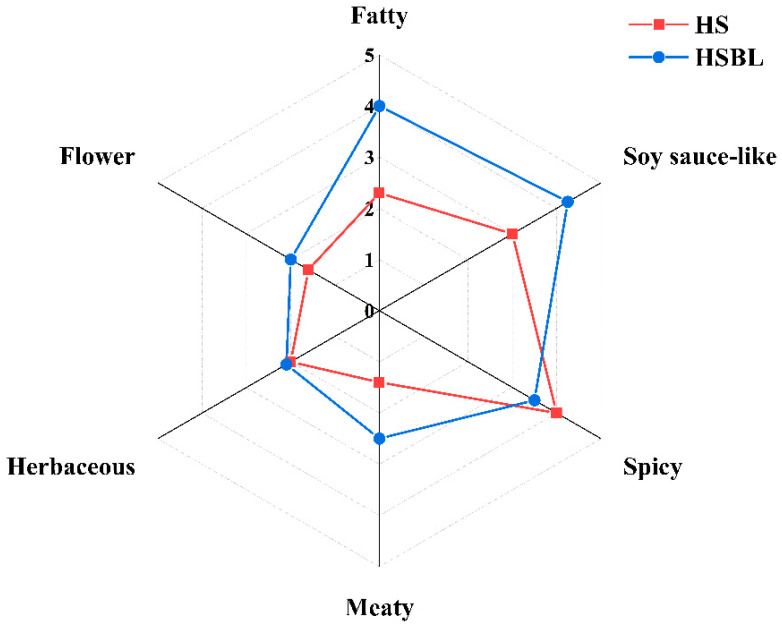
Sensory evaluation of Hotpot seasoning before and after boiling.

According to the sensory evaluation scores of trained sensory experts (Figure 1), the overall aroma profile of HS and HSBL was found to be composed of fatty, spicy, soy sauce-like, meaty, flower, and herbaceous. The sensory evaluation results showed that the aroma characteristics of the HS were significantly different from the HSBL. Although HS and HSBL possess similarities in the sensory properties of spicy, flower, and herbaceous, the sensory evaluation score of the HS in fatty, spicy, soy sauce-like, and meaty was significantly lower than the HSBL. This might be due to the Maillard reaction between the carbohydrate compounds and free amino acids in the HS during boiling, which was consistent with previous studies [13,14]. Additionally, it was found that the sensory properties of spicy in the HS were higher than the HSBL. However, after boiling for 0.5 h, the sensory properties were all improved except for herbaceous. Of them, the sensory evaluation score in meaty, fatty, and soy sauce-like were significantly increased. After boiling for 0.5 h, the intensity of sensory properties of each aroma in the HS was more balanced, and the overall aroma was softer, without any prominent single spicy flavor. This could be a reason for the high preference for HSBL over HS.

### 2.2. The Effect of Boiling on the Composition of Aroma Compounds in Hotpot Seasoning

**Figure 2:**
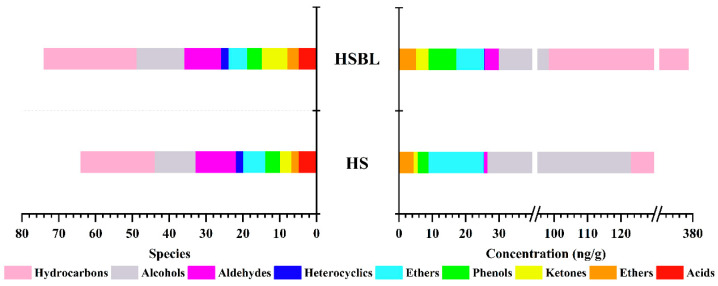
The accumulation diagram of aroma compounds of Hotpot seasoning before and after boiling.

The aroma compounds were extracted from the HS and HSBL by SAFE and analyzed by the SGC/GC×GC-O-MS system. As illustrated in Figure 2, a total of 79 aroma compounds were identified and divided into nine chemical categories, namely, hydrocarbons, aldehydes, esters, acids, ketones, alcohols, ethers, phenols, and heterocyclic compounds. Of them, most are the key aroma compounds of butter, pepper, Chinese prickly ash, and other spices [15,16]. For instance, the primary influencing substances of butter flavor such as nonanal, octanal, linalyl acetate, isobutyl acetate, and 2-amylfuran were detected [17]. Additionally, hydrocarbon compounds were the most abundant in 25 species of HS and HSBL. This result is consistent with previous studies [1].

A total of 64 and 74 aroma compound species were identified in the HS and HSBL, respectively (Table 1). About 60 aroma compounds were in the HS and HSBL, including 19 hydrocarbons, 10 alcohols, three ketones, four acids, two esters, four phenols, two heterocyclic compounds, five ethers, and nine aldehydes. The results showed that the aroma compounds of HS were changed significantly after boiling. Compared with the HS, the species of alkenes, alcohols, esters, and ketones were increased. This might be the reason to the increase in the sensory properties of fatty, meaty, and soy sauce-like enhance in the HSBL [18,19]. Additionally, the species of aldehydes and ethers showed a decreasing trend. This possible reason could be some of the aroma substances might have broken down or reacted with other compounds to produce new compounds during heating [20]. The change in the aroma compound species makes the HS before and after boiling has produced a significant difference, which could be monitored by sensory evaluation. Obviously, the difference between HS and HSBL also could be attributed to the changes in the aroma compound concentrations.

Compared with the HS, the total aroma compound contents significantly increased in the HBLS, and the total relative concentration increased from 277 μg/g to 379 μg/g. Besides ether, alcohols, and acid compounds, the other species’ compound contents were increased, of which the hydrocarbon compounds reached the highest level in the HSBL, especially the terpenes. Terpenes are one of the most structurally diverse secondary metabolites in plants and play a significant role in plant biology [21]. The terpenes in the HS are contributed by the spices. However, there was no significant difference between the species’ compounds in the HS and HSBL. Therefore, it might be possible that the high temperature during boiling causes the release of terpenes further. It is worth noting that the aldehyde contents showed the highest increasing trend of about 4.38 times after the boiling of HS, indicating that aldehyde compounds may contribute more to the aroma of HSBL than HS. Besides, esters and ketones in the HSBL also increased significantly. This result is consistent with previous studies in Chinese foxtail millet (Setaria italica) [8]. The formation of ketones during boiling could be attributed to the oxidative degradation of unsaturated fatty acids [8,22], while ester compounds could be formed from organic acids and aliphatic alcohols, which might also be the reason for the decreased content of acid and alcohol compounds.

### 2.3. The Effect of Boiling on the Aroma-Active Compounds of Hotpot Seasoning

Aroma plays a vital role in determining consumers’ choice, perception, and acceptance of food. It also contributes to 80% of the pleasure in food. Therefore, aroma plays an essential role in food. Generally, the compounds smelled by an olfactory are called aroma-active compounds in food. As summarized in Table 1, there were 30 aroma-active compounds that can be smelled in the HS, and 50 species in the HSBL. The results elucidate that boiling not only changes the composition of the aroma compounds in the HS, but also changes the composition of aroma-active compounds, contributing a special flavor to the HSBL.

The AEDA method was employed to further analyze the effect of boiling on the aroma-active compounds in the HS and HSBL. A total of 56 aroma-active compounds were identified in the HS and HSBL (as shown in Table 1). After boiling, the key aroma-active compound species changed from 30 (HS) to 50 (HSBL). Besides the addition of new aroma-active compounds, a decrease in some aroma-active compounds was also observed from HS to HSBL. Therefore, although the HS and HSBL had the same aroma-active compounds, their FD factor was constantly changing. Of them, β-myrcene (nine, woody), D-limonene (27, citrus), p-cymene (9, spices), and β-caryophyllene (9, spices) were significantly decreased in the FD factor of the HSBL. However, their FD factors were still greater than 1, indicating that these compounds still contributed to the sensory properties of spices in the HSBL, which was consistent with the sensory results. In addition to these compounds, the FD factor of fatty aldehydes was significantly changed during the boiling process of HS. In general, fatty aldehydes are produced by fat oxidation and have a low odor threshold [23], hence, they can be smelled even at low concentrations. These results suggest that fatty aldehydes play an important role in the boiling process of HS. Specifically, the FD factors of (*E*)-2-octenal (243, fatty), (*E*)-2-nonenal (243, fatty), methional (243, roast potato), and (*E*)-2-heptenal (81, fatty) were significantly increased, contributing to the sensory properties of fatty and beef-like flavor in the HSBL [17,24]. Meanwhile, it also explains the obvious increase in the sensory properties of meaty and fatty aroma in the HSBL. Additionally, the FD factors in octanal (81, fatty) and phenylethanal (27, floral) were significantly increased in the HSBL. Other compounds with significant changes in the FD factor included 4-methyl-5-thiazoleethanol (81, nutty), and linalool (243, floral), which are mostly derived from the spice in the HS that contributes to the sensory properties of herbaceous and spicy to the HSBL [15,16].

### 2.4. Quantitation of the Key Aroma-Active Compounds and OAVs

Based on the AEDA analysis results, the standard addition method was adopted to quantitatively analyze 23 aroma-active compounds with FD factor greater than three and verify the contribution of aroma-active compounds to the HS and HSBL. The quantitative results are summarized in Table 2. Of these compounds, linalool had the highest content of 88.76 mg/kg in the HSBL. Besides linalool, 1,8-cineole, linalyl acetate, and D-limonene also had higher content in the HS and HSBL. This result was consistent with the previous study [1].

The contribution of aroma compounds does not depend on their concentration but on OAV. When the OAV is greater than or equal to 1, the compounds are considered aroma-active [25]. Except for butanoic acid, methyl eugenol, α-terpineol, and acetic acid, the concentrations of the other 19 aroma-active compounds were all higher than their perception thresholds. Therefore, these compounds were assumed as key aroma-active compounds in the HSBL. Of them, the OAV of (*E*)-2-octenal was the highest (279), which was consistent with the AEDA results, in which (*E*)-2-octenal showed the highest FD factor. This was followed by methional (266, roast potato) and (*E*)-2-heptaenal (187, fatty). The OAVs of 20 key aroma-active compounds in the HS were greater than or equal to 1. Linalool (42, floral) was the highest OAV compound in the HS, which differed from the HSBL. Notably, methional had the OAV less than 1 in the HS, which might be related to its low and non-detectable content in the HS. This result further confirmed that the boiling process has an effect on the aroma of HS.

### 2.5. Correlation between Key Aroma-Active Compounds and Sensory Properties

**Figure 3:**
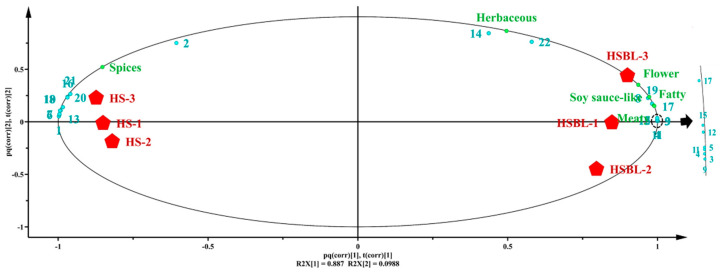
The correlation between sensory properties and important key aroma-active compounds by O2PLS modeling before and after boiling of Hotpot seasoning.

People take the minimum concentration of aroma compounds at the beginning of the smell as a unit to indicate the intensity of aroma (called the threshold). Existing studies have confirmed that high concentrations of aroma components do not always play an important role in odor contribution because odor levels are related to their thresholds [26]. OAVs could accurately reflect the level of odor based on the approximate result from the comparison between OAVs and sensory evaluation scores. Thus, based on the sensory evaluation scores and OAV of 22 key aroma-active compounds, the correlation analysis of these data was performed using the O2PLS model to reveal the relationship between key aroma-active compounds and sensory properties in HS and HSBL, and the results are illustrated in Figure 3. The R^2^ and Q^2^ values represent the interpretation rate and predictive capability of the model, respectively. R^2^X represents the model’s ability to explain the X variable. The closer R^2^ and Q^2^ values are to 1, the better the predictive ability of the model, where R^2^ and Q^2^ were 0.999 and 0.997, respectively, suggesting that OAV of the key aroma-active compounds could be promising to develop a reliable model for six sensory properties evaluation (Q^2^ ≥ 0.50) [27]. The projection distance of the sample reflects the size of the difference between the samples. The farther the sample is, the greater the difference and the more obvious the classification. 

As illustrated in Figure 3, HS and HSBL were far apart and hence, could be grouped into two categories. This result was consistent with the sensory evaluation results. The results indicated that multivariate statistical analysis of the OAV values of the key aroma-active compounds in the samples could characterize the true information of the samples in a better way. Additionally, the sensory properties of meaty, soy sauce-like, and fatty were closely related to the HSBL, indicating that the scores of these three sensory properties contributed to its clustering. Similarly, the distance between the sensory properties of spicy and HS was close, which was also consistent with the sensory evaluation results, indicating that the key aroma-active compounds of HS and HSBL are positively correlated with the sensory evaluation. Therefore, the correlation between the sensory properties and key aroma-active compounds could be determined based on their distribution in the score chart. Acetic acid, thujone, methyleugenol, p-cymene, diallyl disulphide, α-terpineol, β-caryophyllene, β-myrcene, 1,8-cineole, and D-limonene were positively correlated with the sensory properties of spice in the samples. Similarly, methional, nonanal, phenylethanal, ethyl caprylate, 4-methyl-5-thiazoleethanol, (*E*)-2-octenal, humulene, (*E*)-2-heptenal, acetophenone, and linalyl acetate were positively correlated with the sensory properties of flower, meaty, soy sauce-like, and fatty in the samples. This was consistent with the previous sensory evaluations. Notably, α-pinene and linalool were more strongly associated with the sensory properties of herbaceous. This might be due to the interaction between the compound and other compounds or the sample matrix, regardless of its actual behavior in the food matrix or aroma compound mixture [28].

## 3. Materials and Methods

### 3.1. Samples

The Haorenjia brand of HS was purchased from Beijing Yonghui Supermarket and produced by Sichuan Teway Food Group Co., Ltd., Chengdu, China. One thousand hundred grams of ultra-pure water (room temperature 25 °C, KeDa, Shenzhen, China) was heated to boiling point in a constant temperature water bath (set temperature 100 °C, JB Nova, Grant, UK). Then, 500 g HS was cut into small pieces with a volume of about 8 cm^3^, and placed into boiling ultra-pure water to be melted. After the mixture of HS and ultra-pure water was cooked to boiling for 30 min and cooled to room temperature, the HSBL was obtained by removing the solids from the boiled liquid of HS. The HSBL was immediately used for further analysis.

### 3.2. Chemicals

All 23 standard compounds used for the quantification analysis were purchased from Sigma-Aldrich (purity 99.0%, all chromatographic grade), including butanoic acid, acetic acid, thujone, methyl eugenol, p-cymene, α-pinene, diallyl disulphide, α-terpineol, β-caryophyllene, β-myrcene, 1,8-cineole, D-limonene, linalool, methional, nonanal, phenylethanal, ethyl caprylate, 4-methyl-5-thiazoleethanol, (*E*)-2-octenal, (*E*)-2-heptenal, humulene, acetophenone, and linalyl acetate. The internal standard (2,4,5-trimethylthiazole) and n-alkanes (C8–C30) used to calculate the retention index (RI) were of chromatographic grade and purchased from Sigma-Aldrich (St, Louis, MO, USA). Diethyl ether and n-pentane for dilution and preparation of sample were obtained from Thermo Fisher Scientific Inc. (Waltham, MA, USA). High purity helium (99.999% purity) and nitrogen (99.99% purity) were purchased and produced by Beijing Tianlirenhe Trading Co., Ltd. (Beijing, China).

### 3.3. Establishment and Evaluation of Aroma Profile

The sensory evaluation team consisted of 12 experienced sensory evaluators from the molecular sensory laboratory of Beijing Technology and Business University (including 4 males and 8 females, with an average age of 24). The sensory training was carried out before the formal evaluation activities to make the team members more familiar with the sensory evaluation indicators. The sensory evaluation indicators described the roast, meaty, soy sauce-like, onion- and ginger-like, sweet, burnt, sulfur-like, spicy, and fatty, etc. Each index was rated on a scale of 0 to 5, with 0 being tasteless and 5 being very strong. The sensory evaluation was conducted in a blind tasting, i.e., the sensory evaluators did not know the specific information of the samples in advance. The average value of the evaluation results of each sensory descriptor was calculated. The specific sensory operation process was as follows:

(1) Firstly, the sensory evaluation team was asked to conduct the first round of descriptive sensory evaluation, i.e., on the premise of not providing any descriptors to guide their judgment. The sensory evaluation was conducted based on their subjective feelings and only recorded what they smelled.

(2) Secondly, all the participants were allowed to discuss the sensory descriptors and then retained and confirmed the following three types of sensory descriptors: the common description word of the record, the words with the same meaning but different descriptions, and the word can be recalled that you actually smelled from someone else’s description.

(3) Finally, the quantitative descriptive analysis was carried out based on the selected sensory descriptors, i.e., sensory evaluation was conducted on the sample again, and the determined descriptors were scored. Each team member was required to evaluate the same sample three times, and the final score was an average score of the three samples.

### 3.4. Isolation of the Volatiles by Solvent-Assisted Flavor Evaporation (SAFE)

Later, 60 mL of diethyl ether and 30 mL of n-pentane were mixed with the heated, melted HS (25 g) and HSBL (25 g), respectively. Then, 2,4,5-trimethylthiazole (1.013 μg/mL, 5 μL) as the internal standard was added in HS. The mixture was shaken at 180 r/min for 4 h at 4 °C, and the organic phases were separated and collected. The volatile compounds were distilled under a high vacuum by solvent-assisted flavor evaporation (SAFE) [29]. The temperature of the water bath was 40 °C, and the vacuum pressure was less than 5.0 × 10^−3^ Pa. The volatile fractions were cooled and collected with liquid nitrogen. After the resulting fraction was cooled to room temperature, anhydrous sodium sulfate was added for water removal and filtration. The resulting fraction was then condensed to 500 μL by a Vigreux column (50 cm × 1 cm; Beijing Jingxing Glassware Co., Ltd., Beijing, China) in the presence of nitrogen flow. Each sample was then analyzed by the newly switchable GC/GC×GC-olfactometry-mass spectrometry (SGC/GC×GC-O-MS) system.

### 3.5. SGC/GC×GC-O-MS Analysis System

The new SGC/GC×GC-O-MS system was used to analyze the aroma compounds in HS. The system was composed of three parts: gas chromatography (Agilent 8890-5977B, Agilent Technologies, Santa Clara, CA, USA), sniffing detection (Sniffer 9000, Brechbuhler, Schlieren, Switzerland), and solid-state modulator (J&X Technology, Harbin, China). Moreover, the system was equipped with two columns of different polarities for separating the aroma compounds. One column was a polar DB-Wax column (30 m × 0.25 mm × 0.25 μm; J&W Scientific, Folsom, CA, USA), and the other one was a medium-polarity DB-17 ms column (1.85 m × 0.18 mm × 0.18 μm; J&W Scientific, Folsom, CA, USA). The solid-state modulator SSM1800 (J&X Technology, China) was located between two columns and used for heating and cooling. The temperature of the cold zone was −50 °C. The temperature at the inlet and outlet of the heating zone was 70 °C and 160 °C, respectively, and the modulation period was 4 s [30]. The initial column temperature was set at 40 °C and held for 3 min, then increased to 250 °C at a rate of 5 °C/min and held for 5 min. Ultra-high purity helium (purity = 99.999%) was used as the carrier gas at a constant flow rate of 1.2 mL/min, and the split mode was set as splitless. The injector temperature was 230 °C, and the pressure was 15.74 psi. The quadrupole temperature was 220 °C, and the range of MS scanning was set to 29–500 *m*/*z*. The solvent delay was set to 4 min. The ion source was electron impact (EI). The compounds generated electron impact mass spectrum at 70 eV ionization energy and the temperature was set at 230 °C. The sniffing detection was installed at the end of the first column [31]. At least three professional sensory evaluators were allowed to sniff the same sample to ensure experimental accuracy. The suction port temperature was set to 250 °C.

### 3.6. Qualitative Analysis

The SGC/GC×GC-O-MS was employed for the qualitative analysis of aroma compounds in the HS and HSBL by mass spectra (MS), retention index (RI), and the olfactory results (O). The results of MS were compared with the 2017 NIST14 mass spectrometry library, and the preliminary qualitative analysis of the compounds was conducted according to the matching degree and the structure information of the mass spectrometry. Then, the identified compound was reconfirmed by comparing it with the standard RI of the target compound. The RI of each target compound was calculated by the retention times of a homologous series of n-alkanes [32]. Afterward, the specific descriptions of aroma compounds recorded by professional sensory assessors were compared with the aroma descriptions of the identified compounds to identify the compounds accurately. Finally, the standard compound (STD) was used to validate the results. 

### 3.7. AEDA

Aroma-active compounds in the HS and HSBL were identified by AEDA. The volatile isolates in the HS and HSBL were stepwise diluted with diethyl ether and n-pentane (the volume ratio was 2:1) in a multiple of 1:3. Sensory evaluation of each sample was performed by three experienced sensory evaluation team members (two women and one man). The series of separations were submitted to SGC/GC×GC-O-MS using the same GC conditions described above. Each sample was tested three times until the odor of the compound vanished. The flavor dilution (FD) factor was then obtained for each aroma compound according to the corresponding dilution multiple; it was used to indicate the maximum dilution perceived by the aroma compound. The higher the FD value, the more is its contribution to the overall aroma of the sample.

### 3.8. Quantitative Analysis

According to the AEDA results, 23 aroma-active compounds with an FD ≥ 3 were selected and quantified by the standard addition method. Similarly, according to the semi-quantitative analysis results, 23 key aroma-active compounds were divided into two groups. Group 1 consisted of butanoic acid, acetic acid, thujone, methyl eugenol, p-cymene, α-pinene, diallyl disulphide, α-terpineol, β-caryophyllene, β-myrcene, 1,8-cineole, D-limonene, and linalool. Group 2 consisted of methional, nonanal, phenylethanal, ethyl caprylate, 4-methyl-5-thiazoleethanol, (*E*)-2-octenal, (*E*)-2-heptenal, humulene, acetophenone, and linalyl acetate. Then, 2,4,5-trimethylthiazole (5 μL, 1.013 μg/μL) was added to each group as an internal standard for the calibration to the quantification of compounds. The two groups of standard mixtures were stepwise diluted by diethyl ether and n-pentane (the volume ratio was 2:1) in a multiple of 2 to obtain a total of 11 gradients. Finally, the standard curve was prepared by plotting the area ratio of the standard compound to the internal standard and the response ratio of the corresponding concentration. All analyses were conducted in triplicate.

### 3.9. OAVs

The concentration of aroma-active compounds (FD ≥ 3) and their threshold values in the oil were used to calculate the OAVs, better understand the contribution of key aroma-active compounds to HS and HSBL, and their change before and after boiling. OAVs were calculated by dividing the concentration of an aroma compound by its odor threshold. Generally, aroma compounds with OAVs of 1 or more are considered to contribute to the overall aroma of the sample [33].

### 3.10. Statistical Analysis

All experiments were conducted in triplicate, and the data were expressed as the mean ± standard deviation. The mean and standard deviation of all aroma compounds was performed by Microsoft Office Excel 2019. The stack diagram and Venn (VN) diagram were generated by OriginPro 2021b software. The bidirectional orthogonal partial least squares (O2PLS) was performed by SIMCA-P 14.1 software. O2PLS is a multivariate projection method that extracts linear relationships from two data blocks X and Y by removing the so-called structured noise. Noise data refers to the possible deviation or error between the measured value and the real value in the measurement of variables, which will affect the correctness and effect of subsequent analysis operations.

## 4. Conclusions

In this study, aroma compounds in the HS and HSBL were analyzed by the molecular sensory analysis method. According to the results, more prominent sensory properties of meaty, soy sauce-like, and fatty in HSBL confirmed that the boiling process will positively impact the HS. Hydrocarbon compounds were the most abundant species in the HS and HSBL. Combined with the AEDA analysis, the difference in the aroma-active compounds in the HS and HSBL was distinguished effectively. The results showed that 13 aroma-active compounds were significantly affected by the boiling process, including β-myrcene, D-limonene, (*E*)-2-octenal, methional, (*E*)-2-heptenal, and linalool. Furthermore, 23 key aroma-active compounds were identified by the quantitative analysis and OAVs. Of them, (*E*)-2-octenal was the highest OAV compound in the HSBL, followed by 3-methylthiopropionic aldehyde, while linalool was the highest OAV compound in the HS, followed by (*E*)-2-heptenaldehyde, which was obviously different from the HSBL. Furthermore, the correlation between the key aroma-active compounds and the sensory properties of HS and HSBL was explored. Overall, this study will provide a theoretical basis for improving the quality of HS and flavor control in the boiling process.

## Figures and Tables

**Table 1:** Aroma compounds in hotpot seasoning before and after boiling.

No.	Compound	RI ^a^	Odor ^b^	Identification ^c^	Concentration (μg/g) ^d^		FD ^e^	
					HS ^f^	HSBL ^g^	HS ^f^	HSBL ^g^
***Hydrocarbons***								
1	*D*-(+)-α-pinene	997	Minty	MS/RI	ND	1.87 ± 0.034	-	-
2	α-pinene	997	Pine, turpentine	MS/RI/O/STD	0.394 ± 0.024	0.371 ± 0.013	3	3
3	toluene	1008	Paint	MS/RI/O	71.6 ± 5.13	110.0 ± 5.99	-	1
4	sabinene	1095	Pepper, turpentine	MS/RI/O	7.39 ± 0.643	27.5 ± 1.41	-	1
5	myrcene	1138	Balsamic, must, spice	MS/RI/O/STD	1.90 ± 0.051	0.945 ± 0.039	27	9
6	α-phellandrene	1141	Turpentine, mint, spice	MS/RI/O	0.140 ± 0.004	0.855 ± 0.095	-	1
7	1,2-dimethylbenzene	1156	Geranium	MS/RI/O	1.20 ± 0.11	0.849 ± 0.047	1	-
8	α-terpinene	1156	Lemon	MS/RI	0.161 ± 0.029	1.75 ± 0.03	-	-
9	(*S*)-(−)-limonene	1174	Turpentine, wood	MS/RI	ND	0.200 ± 0.045	-	-
10	(+)-limonene	1176	Citrus, mint	MS/RI/O/STD	13.2 ± 0.34	10.0 ± 0.388	81	27
11	β-phellandrene	1184	Mint, terpentine	MS/RI/O	0.809 ± 0.023	6.51 ± 0.303	-	1
12	γ-terpinene	1223	Gasoline, turpentine	MS/RI/O	0.342 ± 0.023	4.26 ± 0.215	-	1
13	β-ocimene		Citrus, green	MS/RI/O	0.087 ± 0.01	0.033 ± 0.007	-	-
14	p-cymene	1244	Solvent, gasoline, citrus	MS/RI/O/STD	0.371 ± 0.034	0.081 ± 0.008	81	9
15	δ-terpinene	1260	Pine, plastic	MS/RI	0.122 ± 0.016	ND	-	-
16	β-caryophyllene	1580	Wood, spice	MS/RI/O/STD	0.456 ± 0.033	0.384 ± 0.026	27	9
17	α-humulene	1649	Wood	MS/RI/O/STD	0.159 ± 0.009	0.950 ± 0.040	3	9
18	germacrene D	1686	Wood, spice	MS/RI/O	0.502 ± 0.075	2.96 ± 0.048	-	1
19	δ-elemene	1711	Wood	MS/RI	0.111 ± 0.012	0.088 ± 0.003	-	-
20	δ-cadinene	1733	Thyme, medicine, wood	MS/RI	0.103 ± 0.01	0.590 ± 0.036	-	-
10	(+)-limonene	1176	Citrus, mint	MS/RI/O/STD	13.2 ± 0.360	10.0 ± 0.388	81	27
21	β-sesquip-hellandrene	1743	Wood	MS/RI	0.016 ± 0.001	0.055 ± 0.005	-	-
22	2,6-di-tert-butyl-p-methylphenol	1876	Mild phenolic camphor	MS/RI	29.3 ± 0.203	13.4 ± 0.086	-	-
23	α-copaene	1481	Wood, spice	MS/RI/O	ND	2.33 ± 0.633	-	1
24	α-zingiberene	1695	Spice, fresh, sharp	MS/RI	ND	1.48 ± 0.029	-	-
26	isocaryophyllene	1953	wood	MS/RI	ND	0.042 ± 0.018	-	-
***Total***					128.4 ± 7.62	187.5 ± 9.52		
***Aldehydes***								
27	methylbutanal	860	Cocoa, almond	MS/RI/O	0.137 ± 0.011	ND	1	-
28	octanal	1262	Fat, soap, lemon	MS/RI/O	0.011 ± 0.003	0.061 ± 0.003	-	1
29	decanal	1472	Soap, orange	MS/RI/O	0.024 ± 0.002	0.038 ± 0.004	1	1
30	benzaldehyde	1492	Burnt sugar	MS/RI/O	0.114 ± 0.014	0.106 ± 0.038	1	1
***Aldehydes***								
31	trans-2-nonenal	1507	Cucumber, fat, green	MS/RI/O/STD	0.497 ± 0.026	2.44 ± 0.113	27	81
32	myrtenal	1599	Spice	MS/RI/O	0.018 ± 0.002	0.356 ± 0.008	-	1
33	methional	1681	Cooked potato	MS/RI/O/STD	-	0.053 ± 0.004	-	243
34	trans-2-octenal		Green, nut, fat	MS/RI/O/STD	0.059 ± 0.005	0.837 ± 0.028	27	243
35	2-phenyl propionaldehyde	1851	Hyacinth	MS/RI	0.014 ± 0.001	ND	-	-
36	p-anisaldehyde	1980	Mint, sweet	MS/RI	0.091 ± 0.006	0.222 ± 0.015	-	-
37	nonanal	1390	Fat, citrus, green	MS/RI/O/STD	0.005 ± 0.001	0.093 ± 0.004	9	81
38	phenylethanal	1608	Hawthorne, honey, sweet	MS/RI/O/STD	0.013 ± 0.001	0.106 ± 0.010	3	27
***Total***					0.983 ± 0.081	4.31 ± 0.223		
***Alcohols***								
39	pentanol	1179	Balsamic	MS/RI	0.098 ± 0.009	0.101 ± 0.014	-	-
40	1,8-cineole	1186	Mint, sweet	MS/RI/O/STD	5.16 ± 0.190	4.15 ± 0.255	9	3
41	(*E*)-furan linalool oxide	1445	Flower	MS/RI/O	ND	0.125 ± 0.025	-	1
42	linalool	1516	Flower, lavender	MS/RI/O/STD	88.8 ± 2.59	58.7 ± 0.905	27	243
43	1-terpinen-4-ol	1575	Turpentine, nutmeg, must	MS/RI/O	0.767 ± 0.112	3.87 ± 0.194	-	1
44	furfuryl alcohol	1626	Burnt	MS/RI/O	0.042 ± 0.005	0.209 ± 0.018	-	1
45	α-terpineol	1664	Oil, anise, mint	MS/RI/O/STD	0.300 ± 0.022	0.162 ± 0.018	3	1
46	methyl eugenol		clove, spice	MS/RI/O/STD	0.272 ± 0.018	0.039 ± 0.003	9	1
47	2-phenyl-2-propanol	1723	Mild green sweet earthy	MS/RI	0.917 ± 0.036	0.290 ± 0.008	-	-
48	p-cymenol	1811	Citrus, must	MS/RI/O	ND	0.095 ± 0.004	-	1
49	phenethyl alcohol	1873	Flower, rose	MS/RI	0.112 ± 0.007	0.046 ± 0.043	-	-
50	elemol	2036	Green, wood	MS/RI	ND	0.054 ± 0.007	-	-
51	limonene glycol	2219	Cool minty	MS/RI	0.016 ± 0.004	ND	-	-
52	4-methyl-5-hydroxyethyl-thiazole	2275	Sulfur	MS/RI/O/STD	0.099 ± 0.009	0.578 ± 0.035	1	9
***Total***					96.5 ± 3.01	68.4 ± 1.53		
***Acids***								
53	butanoic acid	1614	Rancid, cheese	MS/RI/O/STD	0.046 ± 0.004	0.018 ± 0.002	3	1
54	acetic acid		sour	MS/RI/O/STD	0.130 ± 0.011	0.039 ± 0.004	3	1
***Acids***								
55	pentanoic acid	1703	Sweat	MS/RI/O	ND	0.026 ± 0.000	-	1
56	octanoic acid	2017	Sweat, cheese	MS/RI/O	0.019 ± 0.001	0.055 ± 0.004	-	1
57	nonanoic acid	2118	Green, fat	MS/RI	0.018 ± 0.002	0.059 ± 0.005	-	-
58	decanoic acid	2221	Rancid, fat	MS/RI	0.010 ± 0.001	ND	-	-
***Total***					0.222 ± 0.019	0.198 ± 0.015		
***Esters***								
59	ethyl octanoate	1412	Fruit, fatty	MS/RI/O/STD	0.045 ± 0.005	0.063 ± 0.009	3	9
60	linalyl acetate	2176	Sweet, fruit	MS/RI/O/STD	4.24 ± 0.223	4.92 ± 0.241	3	3
61	isobutyl acetate	1029	Fruit, apple, banana	MS/RI	ND	0.139 ± 0.001	-	-
***Total***					4.28 ± 0.228	5.12 ± 0.249		
***Ketones***								
62	6-methyl-5-hepten-2-one	1311	Pepper, mushroom	MS/RI/O	ND	0.006 ± 0.002	-	1
63	4-undecanone	1358	Fruit	MS/RI/O	ND	0.071 ± 0.003	-	1
64	β-thujone	1397	Cedarleaf	MS/RI/O	ND	0.389 ± 0.048	-	1
65	α-thujone	1416	Cedarleaf, thujonic	MS/RI/O/STD	0.014 ± 0.001	0.014 ± 0.001	9	27
66	acetophenone	1617	Must, flower	MS/RI/O/STD	1.20 ± 0.094	3.17 ± 0.206	27	81
67	4-isopropyl-2-cyclohexenone	1642	Spice, caraway	MS/RI	0.021 ± 0.002	0.077 ± 0.009	-	-
68	piperitone	1696	Mint, fresh	MS/RI/O	ND	0.102 ± 0.007	-	-
***Total***					1.24 ± 0.097	3.83 ± 0.277		
***Phenols***								
69	estragole	1637	Licorice, anise	MS/RI/O	0.674 ± 0.103	4.10 ± 0.073	-	1
70	methyl eugenol	1968	Spice	MS/RI/O	0.039 ± 0.05	0.256 ± 0.015	-	1
71	ethyl maltol	1976	Sweet, caramel	MS/RI	2.27 ± 0.680	3.55 ± 0.293	-	-
72	2,4-di-tert-butylphenol	2254	Phenolic	MS/RI	0.260 ± 0.061	0.415 ± 0.065	-	-
***Total***					3.24 ± 0.849	8.33 ± 0.466		
***Ethers***								
73	diethyl sulfide	843	Ethereal, sulfurous	MS/RI/O	0.799 ± 0.128	ND	1	-
74	diethyl disulfide	1185	Onion	MS/RI/O	0.090 ± 0.015	0.067 ± 0.002	1	1
***Ethers***								
75	allyl disulfide	1451	Onion	MS/RI/O/STD	0.126 ± 0.010	0.059 ± 0.005	9	3
76	safrole	1838	Spice	MS/RI/O	0.026 ± 0.003	0.170 ± 0.015	-	1
77	myristicin	2213	Spice	MS/RI/O	0.233 ± 0.033	0.639 ± 0.091	-	1
78	anethol	1971	Sweet, licorice	MS/RI/O	15.3 ± 0.032	7.29 ± 0.296	1	-
***Total***					16.6 ± 0.222	8.23 ± 0.509		
***Heterocyclics***								
79	2-acetylfuran	1474	Balsamic	MS/RI/O	0.031 ± 0.006	0.042 ± 0.002	1	1
80	2-acetylpyrrole	1926	Nutty	MS/RI/O	0.080 ± 0.052	0.169 ± 0.015	-	1
***Total***					0.111 ± 0.058	0.211 ± 0.017		

^a^ Retention index; the actual RI could not exceed ±30 of the library standard value. ^b^ Odor perception sensed at the sniffing port. ^c^ Identification methods of each aroma compound. MS, RI, O, and STD represent being identifying by mass spectra, retention indices, olfactometry, and standard. ^d^ The concentration of aroma compounds in hotpot seasoning before and after boiling relative to the internal standard compounds. ^e^ The FD factors of compounds determined by aroma extraction dilution analysis in hotpot seasoning before and after boiling. ^f^ HS: hotpot seasoning. ^g^ HSBL: hotpot seasoning boiling liquid.

**Table 2:** Concentrations, odor thresholds, and OAV of key aroma-active compounds in hotpot seasoning before and after boiling.

No.	Compounds ^a^	Quota Selected Ion (*m*/*z*) ^b^	Odor Threshold (ng/g) ^c^	Concentration (μg/g) ^d^		OAV ^e^	
				HS ^f^	HSBL ^g^	HS ^f^	HSBL ^g^
1	butanoic acid	88 (60, 73)	50	0.076 ± 0.012	0.018 ± 0.008	<1	<1
2	thujone	152 (110, 95)	0.5	0.014 ± 0.001	0.014 ± 0.001	28	27
3	methional	104 (48, 76)	0.2	ND	0.053 ± 0.004	<1	266
4	nonanal	149 (121, 98)	1	0.005 ± 0.0005	0.093 ± 0.004	3	93
5	phenylethanal	120 (91, 92)	4	0.013 ± 0.001	0.106 ± 0.010	3	26
6	methyleugenol	178 (163, 147)	68	0.272 ± 0.018	0.039 ± 0.003	4	<1
7	p-cymene	206 (119, 134)	11.4	0.371 ± 0.034	0.081 ± 0.008	32	7
8	ethyl caprylate	172 (88, 127)	15	0.045 ± 0.005	0.063 ± 0.007	3	4
9	4-methyl-5-thiazoleethanol	143 (112, 113)	100	0.099 ± 0.009	0.578 ± 0.035	<1	6
10	diallyl disulphide	146 (41, 81)	30	0.126 ± 0.010	0.059 ± 0.005	4	2
11	(*E*)-2-octenal	126 (83, 70)	3	0.059 ± 0.005	0.837 ± 0.028	19	279
12	humulene	204 (93, 121)	160	0.159 ± 0.009	0.950 ± 0.040	1	6
13	α-terpineol	206 (93, 105)	280	0.300 ± 0.023	0.162 ± 0.018	1	<1
14	α-pinene	281 (93, 77)	190	0.394 ± 0.024	0.371 ± 0.013	2	2
15	(*E*)-2-heptenal	112 (41, 83)	13	0.497 ± 0.026	2.44 ± 0.113	38	187
16	β-caryophyllene	204 (93, 133)	64	0.456 ± 0.033	0.384 ± 0.026	7	6
17	acetophenone	120 (105, 77)	36	1.20 ± 0.094	3.17 ± 0.206	33	88
18	β-myrcene	267 (93, 69)	915	1.90 ± 0.051	0.945 ± 0.037	2	1
19	linalyl acetate	196 (93, 121)	1000	4.24 ± 0.223	4.92 ± 0.241	4	5
20	1,8-cineole	154 (139, 111)	1000	5.16 ± 0.190	4.15 ± 0.255	5	4
21	*D*-limonene	170 (93, 79)	1200	13.2 ± 0.340	10.01 ± 0.388	10	8
22	linalool	154 (93, 71)	1082	8.88 ± 2.59	5.87 ± 0.905	42	82
23	acetic acid	60 (43, 45)	50	0.130 ± 0.019	0.039 ± 0.04	2	<1

^a^ Key aroma-active compounds (FD factor ≥ 3) in hotpot seasoning before and after boiling. ^b^ The ions selected for quantitative analysis. ^c^ Odor thresholds were referenced from a book named Odour thresholds. Compilations of Odor Threshold Values in Air, Water, and Other Media (Van Gemert, 2003). ^d^ Mean values of triplicates with standard deviations (SDs). ^e^ OAVs were calculated by dividing the concentrations by the odor thresholds. ^f^ HS: hotpot seasoning. ^g^ HSBL: hotpot seasoning boiling liquid.

## Data Availability

Date of the compounds are available from the authors.

## Sample Availability

Samples of Haorenjia brand of hotpot seasoning are available from the authors.
